# Direct observation of TALE protein dynamics reveals a two-state search mechanism

**DOI:** 10.1038/ncomms8277

**Published:** 2015-06-01

**Authors:** Luke Cuculis, Zhanar Abil, Huimin Zhao, Charles M. Schroeder

**Affiliations:** 1 Department of Chemistry, University of Illinois at Urbana-Champaign, Urbana, Illinois 61801, USA; 2 Department of Biochemistry, University of Illinois at Urbana-Champaign, Urbana, Illinois 61801, USA; 3 Department of Chemical and Biomolecular Engineering, University of Illinois at Urbana-Champaign, Urbana, Illinois 61801, USA; 4 Center for Biophysics and Quantitative Biology, University of Illinois at Urbana-Champaign, Urbana, Illinois, 61801 USA; 5 Institute for Genomic Biology, University of Illinois at Urbana-Champaign, Urbana, Illinois 61801, USA

## Abstract

Transcription activator-like effector (TALE) proteins are a class of programmable DNA-binding proteins for which the fundamental mechanisms governing the search process are not fully understood. Here we use single-molecule techniques to directly observe TALE search dynamics along DNA templates. We find that TALE proteins are capable of rapid diffusion along DNA using a combination of sliding and hopping behaviour, which suggests that the TALE search process is governed in part by facilitated diffusion. We also observe that TALE proteins exhibit two distinct modes of action during the search process—a search state and a recognition state—facilitated by different subdomains in monomeric TALE proteins. Using TALE truncation mutants, we further demonstrate that the N-terminal region of TALEs is required for the initial non-specific binding and subsequent rapid search along DNA, whereas the central repeat domain is required for transitioning into the site-specific recognition state.

## Abstract (web summary)

TALEs are programmable DNA-binding proteins with practical use in genome engineering and synthetic biology. Here the authors use single-molecule fluorescence microscopy to establish that TALE proteins function using two distinct DNA-interaction modes during sequence-specific target search.

## Graphical abstract

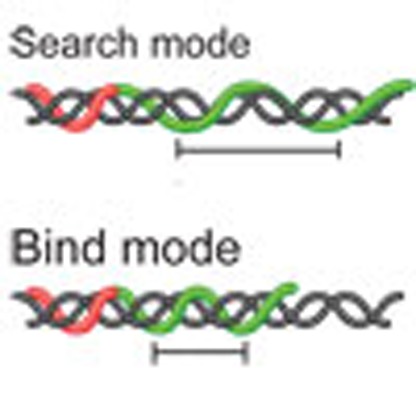

Genome engineering provides an effective framework for the modification of large-scale genomes in a site-specific manner. From a broad perspective, molecular tools for direct editing of genomic DNA could enable the development of improved therapies for treating human disease. Programmable DNA-binding proteins, including zinc-finger proteins^1^ and the CRISPR/Cas9 system^2^, have been established as efficient gene-editing tools in recent years. Transcription activator-like effector proteins (TALEs) have also emerged as a versatile tool for building ‘designer' DNA-binding proteins with high degrees of specificity (Fig. 1)^3^,^4^,^5^,^6^. TALE proteins are characterized by three conserved regions as shown in Fig. 1a: an N-terminal region (NTR) containing the type III translocation system required for secretion, a central repeat domain (CRD) that forms specific DNA contacts and a C-terminal region (CTR) containing nuclear localization signals and an acidic activation domain^7^,^8^. DNA-binding specificity of TALEs is conferred by the CRD, which harbours a series of tandem protein repeats that are typically 34 amino acids in length^7^,^9^. Sequence-specific recognition in the CRD is achieved by the repeat variable diresidues (RVDs), which are amino acids at positions 12 and 13 of each repeat that recognize one of the four nucleobases within the target-binding site^10^,^11^. From this perspective, TALE proteins appear to be distinct from other DNA-binding proteins due to their ‘single repeat to single base' recognition behaviour.

The DNA recognition code for TALE proteins was uncovered in 2009, which has allowed for the rational design of TALE proteins to target virtually any DNA sequence. In particular, simple reprogramming of the RVDs within the CRD tandem repeats allows for the generation of specific DNA-binding proteins. Using this approach, highly specific TALE–nuclease fusion proteins known as TALENs can be constructed by linking nuclease domains (typically the dimeric nuclease *Fok*I) to custom-designed TALE proteins, thereby enabling the direct editing of target sequences in a large genome. Following the initial demonstration of TALENs as artificial nucleases^12^,^13^, TALE proteins have been used in a wide array of applications such as artificial transcriptional activators^14^,^15^,^16^, transcriptional repressors^15^,^17^,^18^, demethylases^19^ and fluorescent probes to study chromatin dynamics in live cells^20^,^21^.

A molecular-level view of sequence-specific DNA recognition by TALEs has been provided by crystal structures of TALEs in both DNA-free and DNA-bound forms (Fig. 1b)^22^,^23^,^24^. Tandem repeats within the CRD exhibit a right-handed superhelical structure that wraps along the major groove of the double helix in B-form DNA. Each repeat in the CRD comprises two α-helices that span residues 3–11 and 14(15)–33 and flank a loop region containing the RVDs (residues 12 and 13). The residue at position 13 specifically interacts with a single base along the DNA template, whereas the residue at position 12 contributes to the stability of the RVD loop. Interestingly, the NTR also displays a right-handed superhelical structure with four continuous repeats that are strikingly similar to the CRD structure but sequence invariant in their binding activity^24^. On binding the target DNA sequence, TALE proteins undergo a conformational change to a more compact form, such that the superhelical pitch is reduced from 60 to 35 Å on binding to target DNA^22^,^25^.

Aside from protein structure determination, the binding behaviour of TALE proteins has also been studied using bulk biochemical assays. Researchers have characterized the binding of TALE proteins to specific and non-specific DNA sequences with varying levels of affinity^24^,^26^,^27^. It has been hypothesized that the NTR of TALEs mediates one-dimensional (1D) sliding on non-specific DNA^24^,^28^; however, there have been no direct observations of the TALE protein search process along DNA templates. Although co-crystal structures and bulk biochemical studies of TALE proteins have revealed a wealth of information regarding specific binding, the dynamic interaction between TALEs and non-specific DNA during the search process is not yet fully understood.

Single-molecule techniques allow for the direct observation of the dynamics of DNA-binding proteins, thereby avoiding the complexities of averaging over heterogeneous dynamics in bulk biochemical assays. In recent years, single-molecule methods have been brought to bear on several biological systems, generally yielding molecular-level insight into structure–function relationships. In particular, the mechanisms of the lac repressor^29^, the DNA repair complex Msh2–Msh6 (ref. 30) and human oxoguanine DNA glycosolase^31^ have been characterized to reveal rapid interrogation of local DNA sequence via 1D sliding mechanisms. Furthermore, single-molecule techniques have demonstrated that several DNA-binding proteins such as EcoRV^32^ and the tumour suppressor p53 (ref. 33) utilize micro-dissociation/reassociation events known as ‘hopping' during DNA target search.

Although facilitated diffusion is a generally accepted method of target location^34^, recent work has shown that not all DNA-binding proteins depend on the canonical ‘sliding' and ‘hopping' mechanisms in locating target-binding sites. Greene and co-workers^35^ utilized a single-molecule-based DNA curtains assay to probe the concentration-dependent dynamics of RNA polymerase, and these studies revealed that RNA polymerase dynamics are dominated by three-dimensional (3D) diffusion at physiological protein concentrations. In related work, Greene, Doudna and co-workers^36^ have shown that the RNA-guided Cas9 endonuclease (used in the CRISPR/Cas9 gene-editing system) similarly locates its target sequence by 3D diffusion. The heterogeneity in fundamental search mechanisms of DNA-binding proteins naturally raises the question: how do TALE proteins locate their target DNA sequences, given that TALEs are structurally distinct compared with other DNA-binding proteins? In this work, we use single-molecule techniques to directly observe the dynamics and search process of TALE proteins along long DNA templates. We investigate the role of TALE subdomains in the search process, and our results suggest that TALE proteins are governed by a two-state ‘search and recognition' mechanism for target recognition along non-specific DNA.

## Results

### TALEs are capable of 1D diffusion along DNA

We studied a series of proteins derived from a parent TALE developed for the editing and correction of the human β-globin gene containing a mutation associated with sickle cell disease^37^. The CRD of the TALE protein contains 21.5 repeats and was engineered using the Golden Gate cloning method. The N-terminal and C-terminal flanking regions were truncated to 208 and 63 amino acids, respectively. We used a 44,898 bp plasmid encoding for a neoaureothin synthesis pathway from *Streptomyces orinoci* as a DNA template^38^. In this work, the plasmid does not contain a binding site for the TALE protein, thereby providing a long double-stranded DNA molecule to observe the dynamics of TALE proteins along non-specific templates.

**Figure 2:**
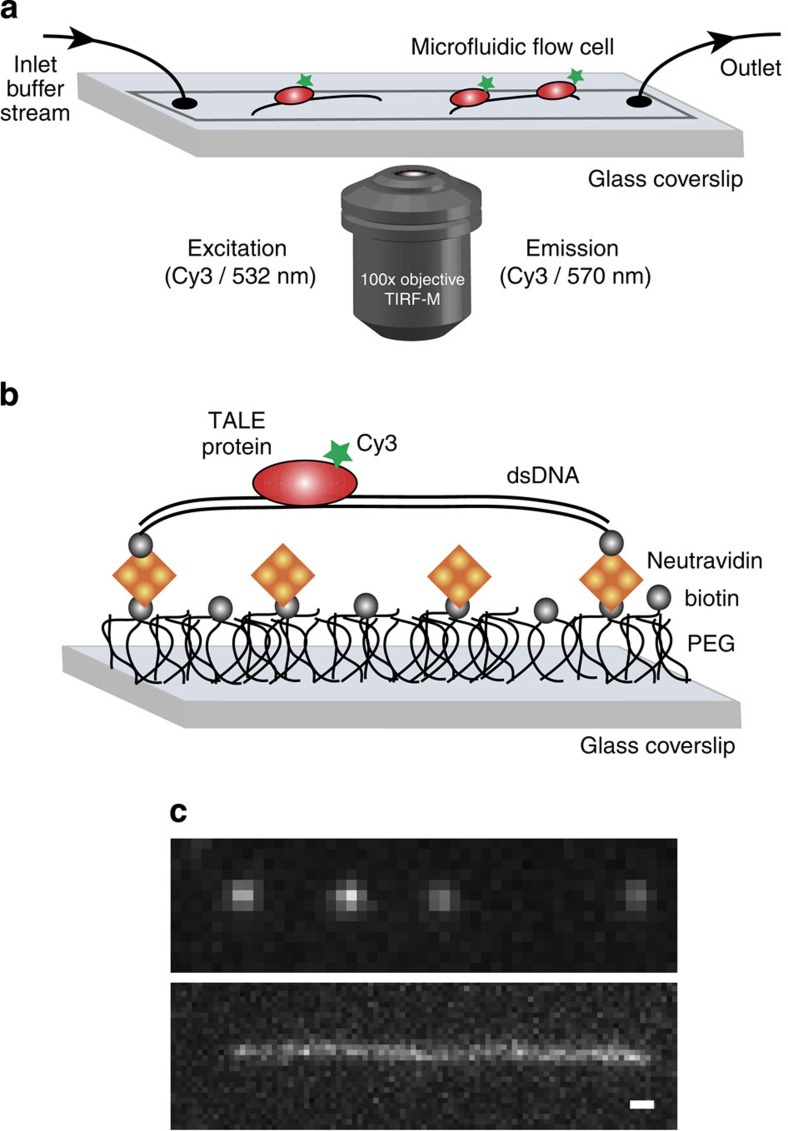
Single-molecule assay for TALE protein dynamics. (**a**) Schematic of experimental setup showing microfluidic flow cell and microscope objective lens for TIRF-M. (**b**) Schematic of dual-tethered DNA assay for TALE protein diffusion. Stretched DNA templates are specifically linked to glass coverslip surfaces via biotin–Neutravidin linkages. (**c**) Top: image of 4 Cy3-labelled TALE proteins bound to template DNA (532 nm excitation/570 nm emission peak). Bottom: image of Sytox Green-labelled DNA template (488 nm excitation/523 nm emission peak). Scale bar, 1 μm.

To directly observe the search mechanism of TALE proteins, we used a single-molecule assay based on dual-tethered DNA imaged using total internal reflection fluorescence microscopy (TIRF-M) (Fig. 2)^39^,^40^. The plasmid is linearized using a single SnaBI restriction site, which facilitates functionalization of both termini and dual tethering to a polyethylene glycolated (PEG) surface via specific chemical linkages (Supplementary Fig. 1). By dual-tethering long linear DNA molecules at high degrees of molecular extension, we are able to generate a field of pristine-binding templates for TALEs while minimizing DNA backbone fluctuations that can obscure spatial localization precision compared with singly tethered molecules stretched in laminar shear flow (Supplementary Fig. 2)^41^. To specifically label TALE proteins with a single organic dye, we used a bioorthogonal aldehyde-labelling scheme (Supplementary Fig. 3)^42^,^43^. In particular, aldehyde-tagged TALE proteins were non-perturbatively labelled with single hydrazine-functionalized Cy3 dyes at the N-terminus. Using this approach, the genetically encoded aldehyde tag and small-molecule dye-labelling method yields fluorescently labelled TALEs with no observed perturbation to binding behaviour relative to the parent protein (Supplementary Fig. 4). During a single-molecule imaging experiment, Cy3-labelled TALEs are introduced into custom-fabricated microfluidic flow cells containing a field of DNA templates. Single-TALE proteins are imaged using a low-light level electron-multiplying charge-coupled device camera (EMCCD), and the spatial positions are determined by fitting a two-dimensional Gaussian function to diffraction-limited spots for the Cy3-TALEs^44^.

**Figure 3:**
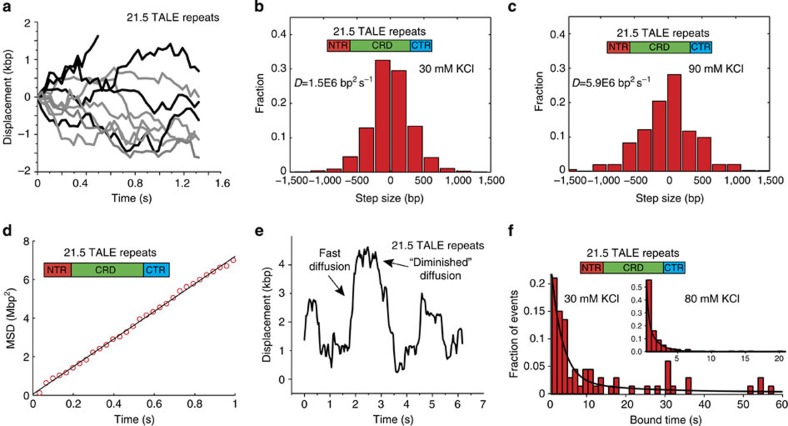
TALE proteins exhibit rapid 1D diffusion along DNA templates. (**a**) Single-molecule trajectories of TALE diffusion events over short timescales (∼1 s) at 90 mM KCl. (**b**,**c**) Histograms of TALE step sizes along non-specific DNA at 30 and 90 mM KCl, respectively. The number of trajectories, *n*, is 50 for both conditions. (**d**) Mean-squared displacement of an ensemble of TALE proteins diffusing along non-specific DNA templates at 90 mM KCl. The values are averages of *n*=50 trajectories. (**e**) Single-molecule trajectory at 90 mM KCl over long timescales (>5 s) shows periods of rapid diffusion interspersed with periods of diminished diffusion. (**f**) Distribution of characteristic TALE bound times at 30 mM KCl and (inset) 80 mM KCl with corresponding double-exponential fits. The number of events recorded, *n*, is equal to 70 and 250 for 30 and 80 mM KCl, respectively.

Using this approach, we directly observed the 1D diffusion of single TALEs along non-specific DNA templates (Fig. 3). The 1D diffusion behaviour of TALEs shows an unbiased directional nature (Fig. 3a), which implies that TALE motion is governed by a stochastic, thermally driven process. In addition, we observed that TALE protein displacement along the axis perpendicular to the DNA templates was minimal and within the spatial precision of the single-molecule assay (Supplementary Fig. 5). We determined distributions of the ‘stepping' behaviour by analysing protein displacement along DNA templates within the camera acquisition time (33 ms) (Fig. 3b,c). Over short timescales, the mean-squared displacement (MSD) versus time for an ensemble of TALE proteins shows a linear relationship (Fig. 3d), which further indicates that TALE motion can be described by a random Brownian process. Although the linearity of MSD versus time provides evidence that TALE dynamics can be described by 1D diffusion, we used a covariance-based estimator method (CVE) to quantitatively determine the apparent 1D diffusion coefficients for single-TALE proteins^45^, which has been shown to minimize localization errors in single-molecule trajectories. Previous studies on TALE proteins have rationalized molecular structure to assess whether TALEs are capable of 1D diffusion along DNA^28^, which has led to some conflicting hypotheses on TALE dynamics. Our results, however, directly show that TALE proteins are capable of utilizing a dynamic search process based on 1D diffusion, which effectively reduces the dimensionality of target sequence search.

### TALEs slide and hop in search of their target

Facilitated diffusion has long been proposed as a method to reconcile the ability of DNA-binding proteins to locate their target sites orders of magnitude faster than the theoretical limit imposed by 3D diffusion^34^,^46^. In the classic model of facilitated diffusion for DNA-binding proteins, non-specific binding is followed by successive phases of protein ‘sliding' and/or ‘hopping'^47^. During 1D sliding, a protein molecule is thought to remain in constant contact with a DNA backbone, following a rotationally coupled trajectory along the DNA helix^48^. In contrast, hopping generally involves brief periods of protein dissociation from the DNA backbone, such that counterion condensation can occur during the unbound state. Following a brief period of unbinding, the protein is able to quickly rebind at the initial location of dissociation or a short distance away (less than the persistence length of the DNA). Generally speaking, the frequency of ‘micro' dissociation/reassociation events is influenced by the affinity of proteins for non-specific DNA, which is governed by electrostatic interactions. Therefore, an increase in ionic strength is expected to increase the energetic barrier for rebinding, thereby increasing the hopping frequency and apparent 1D diffusion speed^48^.

We aimed to determine whether TALE proteins exhibit 1D sliding or hopping by modulating the salt concentration in the single-molecule assay. By comparing the apparent 1D diffusion coefficient for the 21.5 repeat TALE determined using CVE analysis^45^ at low and high salt concentrations (Fig. 3b,c, respectively), we observe that the 1D diffusion coefficient increases with increasing salt concentration. In particular, we find a approximately fourfold increase in the apparent 1D diffusion coefficient from 30 mM KCl (1.5E6 bp^2^ s^−1^) to 90 mM KCl (5.9E6 bp^2^ s^−1^), which strongly suggests that full-length TALEs utilize a hopping mechanism, at least in part, as they diffuse along DNA templates. Nevertheless, given the relatively fast diffusion speeds of TALE proteins along DNA templates, it appears unlikely that TALEs utilize a pure hopping mechanism for target search. Hopping events involve both dissociation and reassociation of protein to DNA, and from this perspective, it is possible that pure hopping events could yield 1D diffusion that is apparently slower than pure sliding events. In the latter case, the protein remains in constant contact with the DNA during its 1D motion, for example, in the case of *lac* repressor search dynamics as discussed by Winter *et al.*^49^. Moreover, other DNA-binding proteins that appear to utilize a pure hopping mechanism exhibit much slower 1D diffusion compared with TALEs^33^,^50^, while those proposed to utilize a pure sliding mechanism appear to diffuse faster than TALEs^31^. Finally, as a control experiment, we probed for any putative correlation between the apparent TALE protein diffusion coefficients with the relative extension of the DNA templates. However, we observe no dependence of the TALE diffusion coefficient on DNA extension in our experiments (Supplementary Fig. 6).

### TALEs utilize a multi-state kinetic process for sequence search

The ability of a DNA-binding protein to rapidly locate its binding site and remain stably bound is central to the function of these proteins. The combination of rapid search and stable binding to a target site requires a substantial free energy minimum for the specifically bound state compared with the non-specifically bound state. Interestingly, this seemingly contradictory requirement confounds the observed rapid 1D diffusion of TALEs and other DNA-binding proteins. How can a homogenous, low energy landscape necessary for rapid 1D diffusion be reconciled in the context of large energetic traps required for stable, specific binding along DNA^51^? The ‘search-speed/stability paradox' can be resolved by considering a two-mode (or even three-mode) mechanism for the search process^52^. In the first mode (that is, the search mode), a DNA-binding protein experiences a low (∼1–2 k_B_T) and homogenous energy landscape during 1D sequence search. In the second mode (that is, the check or recognition mode), the DNA-binding protein experiences a rough energy landscape with a substantial free energy minimum at the target site, thereby enabling specific binding and transiently halting the dynamic motion of 1D diffusion. To realize these different modes, it is thought that DNA-binding proteins delegate responsibility to distinct subunits and/or undergo conformational changes to switch between ‘search' and ‘recognition' modes^33^,^53^.

With this framework in mind, we sought to understand how the TALE search process could be understood in the context of a multi-state search mechanism. We conducted a series of single-molecule experiments with reduced laser illumination intensity, which effectively extends the fluorescence photobleaching lifetimes of DNA-bound TALEs to tens of seconds. In this way, we are able to observe long timescale TALE search and binding events. Strikingly, single-molecule trajectories show a large degree of heterogeneity in TALE protein dynamics (Fig. 3e). In these trajectories, TALEs diffuse rapidly (on average) in one direction along the DNA substrates, followed by a period of ‘diminished' diffusion, during which the protein remains constrained to a relatively short region of DNA and exhibits a nearly stationary motion with extremely slow or no diffusion, as determined within the spatial resolution of our single-molecule assay (see Methods). On the basis of these observations, it is clear that TALE protein behaviour during the arrested phases is essentially static compared with the interspersed periods of rapid diffusion. We note that we exclude periods of apparent pausing in our calculations of diffusion coefficients. However, it is possible that short duration ‘checking' events occur on timescales equal to or faster than our data acquisition, which would influence apparent 1D diffusion coefficients.

In a separate set of experiments, we studied the binding of quantum dot-labelled TALEs on DNA templates containing a single-binding site to verify that the TALE construct was indeed capable of stable and specific binding to the target site (Supplementary Fig. 7). We observed a marked preference for specific TALE binding to the target site on titrating non-specifically bound TALEs from the DNA substrates, indicating that the TALE construct is capable of stable and specific binding to the target site.

We next determined the characteristic binding times of TALE proteins on DNA templates at low and high ionic strengths (Fig. 3f and inset). As expected, the average binding lifetime of TALEs decreases as ionic strength is increased. Interestingly, the distributions of characteristic binding times are not well described by a single-exponential fit for both low and high salt concentrations. In contrast, binding time distributions for full-length TALEs at 80 mM KCl are reasonably well fit to a double-exponential fit, with characteristic long and short binding times of 0.67±0.09 and 4.40±2.24 s, respectively. At low salt concentrations (30 mM KCl), however, there is a broad distribution of long timescale binding events. This behaviour appears to be indicative of a complex interplay between the ‘search' and ‘recognition' modes at low ionic strength, with highly variable frequency and duration of these events within each trajectory. Taken together, the marked heterogeneity in single-molecule trajectories combined with the apparent multimodal distribution of characteristic binding times suggest that TALEs utilize a multi-state model for target search and recognition. Clearly, the distribution of bound times for the full-length TALE protein is not well described by a single-exponential decay. To analyse the data, we use a double-exponential function as a good first approximation to capture the behaviour of the second, longer binding mode. It is possible that the observed binding lifetimes for long-time events are likely distributed with a large degree of complexity, which can be attributed to the heterogeneous interplay between the ‘bind' and ‘check' modes of the target search mechanism.

Recently, other DNA-binding proteins such as tumour suppressor p53 have been observed to utilize multimodal search mechanisms^33^. These proteins are distinctly multimeric, wherein the multi-domain protein structures are capable of ‘delegating responsibility' for different modes of search and recognition^33^. However, TALE proteins are monomeric, apparently lacking the requisite multi-domain structure for task delegation. Despite their monomeric structure, however, TALEs clearly satisfy the ‘search-speed/stability' paradox. On the basis of these observations, we further aimed to understand the TALE search process in the context of the subdomains within the TALE polypeptide chain.

### TALE NTR is critical for non-specific binding and sliding

A recent crystallographic study of a TALE protein partially revealed the structure of the NTR^24^, which is known to be necessary for full activity of TALEN systems. The NTR possesses a striking degree of structural similarity to the tandem repeats in the TALE CRD that contain RVDs. The NTR, however, does not exhibit nucleotide specificity^24^, though it contains additional positively charged amino acids and an ability to bind DNA non-specifically. For these reasons, the NTR was identified as a nucleation site for TALE binding and a potential vehicle for mediating 1D sliding of TALEs, a model which could explain the multimodal dynamics of TALE search and recognition^28^.

**Figure 4:**
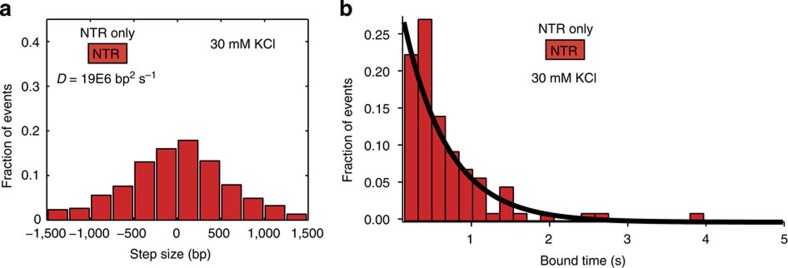
Dynamics of TALE truncation mutants along DNA templates. (**a**) Histogram of step size distribution for NTR diffusion along DNA. The number of trajectories, *n*, is 50. (**b**) Distribution of bound times for NTR truncation mutant on DNA templates, which is described by a single-exponential decay. The number of events recorded, *n*, is equal to 80.

To understand the function of the NTR and its contribution to the overall mechanism of TALE sequence search, we generated a fluorescently labelled TALE NTR truncation mutant, wherein the CRD and CTR are removed and only the NTR remains intact. The NTR truncation mutant was specifically labelled with Cy3 dye using the bioorthogonal aldehyde–hydrazine-labelling method discussed above, followed by direct observation of the DNA search dynamics using our single-molecule assay (Fig. 4). We observed that the TALE NTR diffuses rapidly along DNA templates with an average 1D diffusion coefficient ∼12 × larger (1.5E6 bp^2^ sec^−1^ for the full-length 21.5 repeat TALE and 19E6 bp^2^ s^−1^ for the NTR) than full-length TALEs under the same conditions (Fig. 4a). In general, we found that the TALE NTR displays a lower overall affinity for non-specific DNA compared with full-length TALEs (Supplementary Fig. 4e), which enabled acquisition of single-molecule diffusion data up to salt concentrations of 30 mM KCl. Nevertheless, the TALE NTR showed no significant change in 1D diffusion speed as a function of salt concentration (Supplementary Fig. 8a,b), which starkly contrasts to the strong salt dependence of the 1D diffusion coefficient for full-length TALEs. The salt concentration invariance of the 1D diffusion coefficient for the TALE NTR suggests that the NTR, unlike full-length TALEs, exhibits 1D diffusion via a pure sliding mechanism.

We further measured the characteristic bound times of the TALE NTR truncation mutant and compared these data with the bound time distributions for the full-length TALE (NTR+CRD+CTR, 21.5 repeat) at 30 and 80 mM KCl (Fig. 4b, inset). Unlike the full-length TALE construct, the distribution of bound times for the NTR fit to a single-exponential decay function, which is suggestive of a single-binding mode. The characteristic binding time of the NTR truncation mutant (0.51±0.02 s) is similar to the short timescale binding mode for the full-length TALE at 80 mM KCl, which indicates that the division of labour between the NTR and CRD+CTR domains may be more pronounced at higher ionic strengths. The bound time distribution for TALE NTR further supports the model in which the NTR drives the initial non-specific binding and rapid search activity of TALEs. In this model, the NTR facilitates exploratory search events, whereas the CRD acts as a molecular ‘brake' while checking local DNA sequences and mediating longer duration binding events that can vary in frequency.

Next, we generated a fluorescently labelled TALE truncation mutant containing only the CRD and CTR and lacking the NTR. We observed that this truncation mutant showed no appreciable non-specific binding or search along single DNA templates, even at very low salt concentrations, which is consistent with previous bulk biochemical studies^24^. Overall, our results show that the TALE NTR exhibits fast 1D diffusion along DNA templates, which provides further evidence for NTR serving as a nucleation site for non-specific binding and a vehicle to carry out 1D sliding along non-specific DNA in the context of full-length TALEs^24^. Furthermore, the absence of binding for the CRD+CTR mutant is suggestive of a delicate interplay between the NTR and CRD during the search process, wherein the NTR plays a critical role in maintaining the contact between TALE protein and the DNA template.

### CRD length directly impacts TALE search process

Both naturally occurring pathogenic TALEs and custom-designed TALE(N)s for genome engineering applications exhibit variable size DNA recognition sites typically ranging from 5 to 30 base pairs. To design a TALE protein with a high degree of specificity, it may, therefore, seem logical to generate a TALE construct with a large number of repeats in the CRD, which would tend to maximize sequence specificity. Recent studies, however, have shown that increasing the number of repeats and the corresponding size of the binding site for a TALEN protein generally increases the error rate for nuclease activity^26^,^54^. Furthermore, a computational study on the binding energy contributions of each portion of the canonical repeats revealed that very little overall binding energy is conferred by the actual RVD responsible for sequence specificity^55^. It has been hypothesized that the increased prevalence of off-target binding with increasing CRD length arises from a number of factors, including overall DNA-binding affinity and dephased DNA–protein interactions at locations far from the NTR^26^,^54^. Indeed, understanding the sources of TALE off-target effects is critically important for developing efficient gene-editing tools. For these reasons, we sought to explore the relationship between the number of repeats in the TALE CRD and search dynamics.

**Figure 5:**
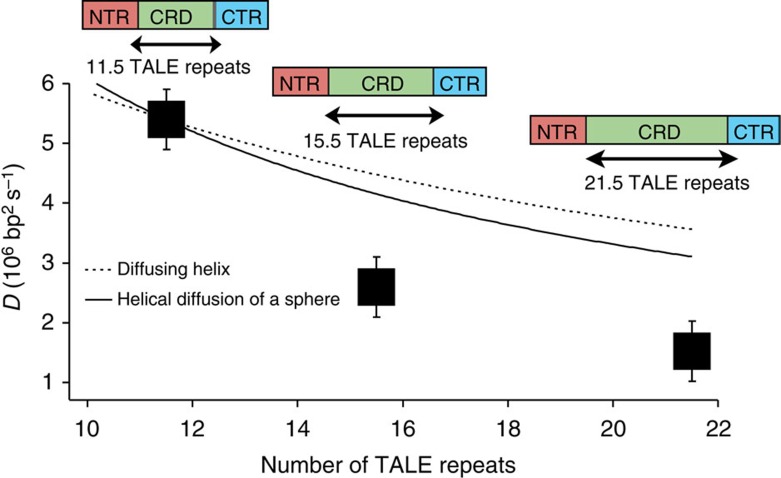
Effect of CRD size on 1D diffusion coefficient of TALE proteins. TALEs with 11.5, 15.5 and 21.5 central repeats were generated and their apparent 1D diffusion coefficients were determined at 30 mM KCl (square symbols). Experimental data are compared with two different hydrodynamic models of diffusion that consider protein conformation as a diffusing helix and a spherical protein tracking the DNA helix, shown by the dashed and solid lines, respectively. Error bars represent the s.d. in the apparent 1D diffusion coefficients.

We generated two fluorescently labelled full-length TALEs containing 11.5 and 15.5 repeats, which are based on the parent 21.5 repeat TALE construct used in this work. Following protein expression, purification and specific labelling with Cy3 dye, we studied the DNA search dynamics of these proteins using our single-molecule assay (Fig. 5). We observe that TALEs with smaller numbers of repeats are able to diffuse along DNA templates faster compared with larger TALEs, with smaller TALE proteins generally showing larger 1D diffusion coefficients. We next sought to understand the potential source of the increased search speeds for smaller TALE constructs. On adding additional repeat domains in the TALE CRD, the length of the superhelical structure of the TALE protein in the specifically bound state effectively increases. From this viewpoint, it is possible that additional repeat domains could simply increase the hydrodynamic drag of experienced by TALEs diffusing along DNA.

To understand the physical mechanisms behind TALE diffusion, we considered a hydrodynamic drag model based on a rigid helical body undergoing 1D diffusion in a direction parallel to the long axis^56^. Using this model, we estimated 1D diffusion coefficients for TALE proteins with variable length CRDs using the shortest TALE protein (11.5 repeats) as a reference. Interestingly, the observed decrease in TALE 1D diffusion coefficients is not quantitatively described by the rotationally coupled hydrodynamic drag model for a helical structure. We also considered a model in which the NTR remains in constant contact with the DNA, tracking the helical structure closely, while the CRD+CTR portions of the protein are pulled along in a less-ordered globular conformation. In the context of this alternative rotationally coupled model for 1D diffusion, the experimental data are similarly not well described by the model. In both cases, the data show a more substantial slowdown in 1D diffusion than predicted by either model based primarily on hydrodynamic drag. We believe that these differences can be accounted for by considering the two-state model for DNA sequence search by TALE proteins (that is, balance between the search mode and recognition mode) and/or the increased role of electrostatic interactions of positively charged residues at positions 16 and 17 within each repeat.

## Discussion

DNA-binding proteins with sequence-specific recognition domains face a daunting task: to search through millions of non-specific sequences for their unique target site. The complex process of DNA search and recognition is further complicated by a fairly homogeneous energetic landscape for specific and non-specific sites. In other words, a combination of rapid dynamics and stable binding to a target site is required for efficient search. To accommodate these seemingly disparate behaviours, a two-state model for sequence-specific search has been proposed for DNA-binding proteins^51^,^57^. Our single-molecule experiments show that TALE proteins exhibit two distinct modes of dynamic behaviour during DNA search, which can be rationalized in the context of TALE protein subdomains and a multi-mode search model. These results have provided, to our knowledge, the first direct observation of TALE search dynamics along DNA templates and the identification of a two-state model of sequence search for a monomeric protein.

The structure of TALE proteins is distinct among the large and diverse family of DNA-binding proteins. TALEs do not possess the common canonical elements such as helix-turn-helix or leucine zipper motifs found in other DNA-binding proteins capable of sequence-specific recognition. Structural studies of TALEs bound to target sites on DNA have revealed a superhelical conformation in the CRD that tracks the major groove of DNA and have also identified the molecular contacts that impart the characteristic base pair specificity of TALEs^22^,^23^. However, the mechanism for TALE interaction with non-specific DNA is generally less clear. What is the molecular conformation of TALE proteins in the search and recognition states? The DNA-free TALE crystal structure indicates that TALE proteins can exist in a looser solenoid conformation with a larger superhelical pitch compared with TALEs bound to target DNA^55^. Indeed, the conformational elasticity of TALEs has been reported in several experimental and computational studies^22^,^25^,^55^,^58^,^59^, wherein a nearly twofold decrease in the superhelical pitch of the TALE CRD is observed on binding to a specific DNA sequence. Moreover, it is known that positively charged patches along the inner surface of the CRD contribute a substantial amount to the non-specific binding energy of TALEs to DNA^22^,^24^. Crystallographic and computational studies have shown that residues G14, K16 and Q17 in each repeat within the CRD confer the majority of the binding energy to the specifically bound state^22^,^23^,^26^,^55^,^58^. Computational studies have also shown that the RVDs exhibit minimal fluctuations and contribute a relatively small amount to the overall binding energy of target-bound TALEs^55^. These findings suggest that TALE sequence specificity is mainly achieved through negative discrimination arising from prohibitive steric and electrostatic clashes rather than from a dominant energetic positive discrimination mechanism^55^.

**Figure 6:**
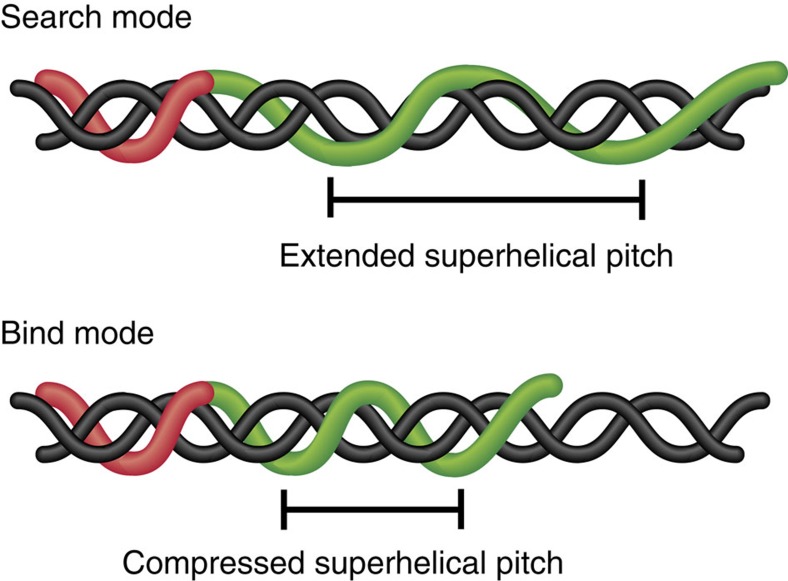
Schematic of two-state model for TALE protein diffusion along DNA. TALE proteins undergo a conformational change from the ‘search' to ‘bind' mode on encountering target DNA. In the ‘bind' mode, the TALE protein is tightly bound to the major groove of a DNA template with non-covalent interactions forming along the phosphate backbone and a superhelical pitch very close to that of B-form DNA. In the ‘search' mode, the protein assumes a looser conformation with a larger superhelical pitch, analogous to the structure DNA-free TALEs^22^. In either mode, the NTR remains in a nearly identical conformation to facilitate 1D sliding motion along DNA.

On the basis of these observations, we conjecture that TALE protein conformation dynamically transitions between a search state and a recognition state that show differences in the superhelical conformation in the CRD (Fig. 6). The search process begins when the TALE NTR initiates binding to non-specific DNA and subsequently facilitates 1D motion along non-specific DNA. In the search mode, the superhelical structure in the CRD is in a looser conformation compared with the recognition mode. In this way, an extended superhelical pitch in the search mode would render molecular contacts between the CRD and phosphate backbone out of register, thereby minimizing strong electrostatic interactions between the CRD and DNA backbone and enabling rapid sliding/hopping of the protein along DNA. During the search process, a TALE protein attempting to transition to the recognition mode with non-target DNA would experience steric and electrostatic clashes that would tend to destabilize the complex. On encountering the correct target sequence, however, the requisite molecular contacts can form between the CRD and the DNA backbone, thereby effectively aligning RVDs and other residues in the CRD array for energetically favourable interactions. In the context of our single-molecule experiments, the transition from the search to recognition mode is borne out by the appearance of a dynamically arrested state in 1D diffusion trajectories.

TALEs utilize a combination of sliding and micro-dissociation/reassociation events known as ‘hops' during the DNA search process, as evidenced from the ionic strength dependence of full-length TALE diffusion. In living cells, where DNA molecules are crowded and coated with accessory DNA-binding proteins, it is necessary for any efficient search strategy to handle complex cellular environments^53^,^60^,^61^. From this perspective, it appears logical that full-length TALEs are capable of hopping, which could enable effective bypass of obstacles bound to the DNA substrates during the search process. Overall, this mechanism would prevent TALE proteins from becoming corralled within a short region of DNA between two obstacles or otherwise engaging in a fruitless or inefficient sequence search. While the TALE NTR appears to utilize a pure sliding mechanism for 1D diffusion, it generally exhibits comparatively short periods of 1D diffusion (generally <1 s, even at low salt concentrations), which could allow for frequent hops utilized by full-length TALEs. The contrasting behaviours of the full-length TALEs and the NTR truncation mutant suggest that there is an intricate interplay between the roles of TALE protein subdomain behaviour during the search process.

TALE proteins can be custom-designed to target DNA sequences of variable length. TALENs designed to target longer DNA sequences have been shown to elicit higher error rates, yet the reasons for this counterintuitive target size/binding efficiency relationship are not fully clear. Our results show that TALE protein diffusion significantly slows down on increasing the size of the target DNA sequence; however, the apparent slowdown in diffusion rates cannot be fully reconciled in the context of a rotationally coupled hydrodynamic drag model. Nevertheless, a two-state model for TALE sequence search can be used to rationalize higher error rates for TALEs with larger CRDs (that is, TALEs targeting longer DNA sequences). If TALE proteins with larger CRDs tend to check local sequence with a higher frequency compared with TALEs with smaller CRDs, then the larger TALE proteins would show a ‘slowdown' in 1D diffusion beyond that given by simple hydrodynamics. In other words, it is possible that TALE proteins with larger CRD domains transition from a search to recognition mode with higher frequency compared with shorter TALE constructs. From this perspective, one could rationalize that TALEs designed to target longer DNA sequences may probabilistically recognize off-target sites at a higher apparent rate than TALEs with smaller CRDs.

From a practical standpoint, genomic engineering applications based on TALE proteins have skyrocketed in recent years. TALEs have been used as therapeutics for the treatment of disease, for genome engineering of a wide array of organisms ranging from plants to livestock to humans and for the development of new fundamental biological models. Despite the flurry of activity in designing new TALE systems, however, the fundamental search dynamics of these proteins has been unclear. To this end, our results provide a molecular-level view of the dynamic search process of TALEs. Achieving a fundamental understanding of the TALE search process could potentially enable the development of improved TALE proteins for genome engineering applications, as new molecular tools such as TALENs with minimal off-target effects are critically needed for the development of improved therapeutics for the treatment of human disease.

## Methods

### Cloning of substrate DNA

The plasmid containing no TALE-binding sites, ‘Target receiver plasmid' (Tar-rec), is a 44,898-bp plasmid (unpublished) containing neoaureothin synthesis pathway from *Streptomyces orinoci*. The plasmid containing a single TALE-binding site, SM1x, was constructed from Tar-rec by the introduction of a fragment containing the TALE target sequence flanked at its 3′ end by a *LacZ* gene. To this end, Tar-rec was linearized with PspXI enzyme. The *LacZ* gene was amplified from pNEB193 plasmid (NEB), where a forward primer contained the TALE-binding 22-nucleotide sequence at its 5′ end. The amplified fragment was spliced with the linearized vector using the Gibson Assembly method. The resulting SM1x plasmid contained the single TALE-binding site ∼11 kb away from the SnaBI restriction site used for double tethering of DNA on a glass slide.

### Preparation of DNA constructs

The plasmid was purified using the Plasmid Midi Kit (Qiagen) and linearized with SnaBI enzyme (NEB) for 3 h, followed by further purification using phenol–chloroform extraction and ethanol precipitation. The purified plasmid was subjected to the 3′–5′ exonuclease activity of T4 DNA polymerase (NEB) to create the 5′ overhangs at both ends of the linearized DNA. Ten microgram of DNA was treated with five units of T4 DNA polymerase in NEB buffer 2 supplemented with BSA for 1 h at 25 °C. The reaction was stopped with 1 μl 20 mM dCTP and the enzyme was heat deactivated at 75 °C for 20 min. The exposed 5′ overhangs were used to sequence specifically anneal 3′-biotinylated oligonucleotides. First, the oligonucleotide with the sequence cagcagttcaacctgttgatagtac/3BioTEG/ (IDT) was annealed in 50 × molar excess to the substrate DNA by heating the mixture at 90 °C for 5 min and gradually cooling to 4 °C. The second oligonucleotide with the sequence tacgtgaaacatgagagcttagtac/3BioTEG/ was subsequently hybridized in the microfluidic flow chamber, as described below.

### Preparation of TALE constructs

*Cloning of tSCA21.5 and Naldt-tSCA21.5*. The gene encoding for the untagged TALE protein (tSCA21.5) was assembled using the Golden Gate cloning method^62^ (Addgene TALEN Kit #1000000024). For this purpose, repeats 1–10 of the TALE were assembled into the pFUS_A30A vector, repeats 11–20 into the pFUS_A30B vector and repeat 21 was inserted into the pFUS_B1 vector using the BsaI-HF (NEB) and T4 DNA ligase (NEB) by cycling the temperature 10 times between 5 min at 37 °C and 10 min at 16 °C, after which they were treated with Plasmid-Safe nuclease (Epicenre Biotechnologies). The DH5α (Cell Media Facility at UIUC) transformants were checked for proper assembly using restriction digestion of the purified (Qiagen) plasmids, and together with the plasmid coding for the last half-repeat pLR(NG), further assembled into a specifically engineered destination vector, pET28-GG-TALE using BsmBI (Fisher Scientific) and T4 NDA ligase. The destination vector contained an N-terminal His-tag and flanking NTR (208 amino acids) and CTR (63 amino acids) of the TALE as well as the BsmBI sites corresponding to the kit BsmBI sites (Supplementary Fig. 7a). For fluorescent tagging of the TALE, we modified the original plasmid pET-tSCA21.5 with an oligonucleotide insert encoding N-terminal LCPTSR hexapeptide (aldehyde tag^42^,^43^) upstream of the His-tag (Supplementary Figure 9a). To this end, the plasmid was amplified in fragments containing the insert, and assembled using the Gibson Assembly Kit (NEB).

*Cloning Naldt-tSCA15.5 and Naldt-tSCA11.5*. To construct TAL effectors shortened to the first 15.5 and 11.5 repeats, we first engineered a destination vector pET28-Naldt-GG-TALE, which contains the aldehyde tag upstream of the His-tag, using the Gibson Assembly Master Mix (NEB). Naldt-tSCA15.5 and Nald-tSCA11.5 were assembled using the Golden Gate cloning method^62^ into the destination vector pET-NaldT-GG-TALE. To this end, repeats 1–10 were assembled into vector pFUSA, repeats 11–15 were assembled into pFUS-B5, and repeat 11 was inserted into vector pFUS-B1 using the BsaI-HF and T4 DNA ligase by cycling the temperature 10 times between 5 min at 37 °C and 10 min at 16 °C, after which they were treated with Plasmid-Safe nuclease. The DH5α transformants were checked for proper assembly using restriction digestion of the purified plasmids. pFUSA containing the first 10 repeats was assembled with either pFUS-B5 (for pTALE15.5) or pFUS-B1 (for TALE11.5) together with the plasmid coding for the last half-repeat into the pET28-GG-TALE vector using BsmBI and T4 NDA ligase.

*Cloning of Naldt-NTR*. To create the NTR truncation mutant, we digested pET-Naldt-tSCA21.5 with SacII and HindIII, and inserted the sequence: CAAAGCGTGGTGGCGTGACCGCGGTGGAAGCGGTCCATGCCTGGCGTAATGCGTTGACGGGCGCCCCCCTGAACTAAGTCAGATAACCGGATACAGACAAGCTTGCGGCCGCACTCGAGCACCAC (Supplementary Fig. 7b) synthesized as a gBlock Gene Fragment (IDT), using the Gibson Assembly Master Mix (NEB) (Supplementary Figure 9b).

*Cloning of Naldt–CRD–CTR*. DNA oligonucleotide primers (IDT) containing BsmBI sites corresponding to BsmBI sites from previous destination vectors were used to PCR amplify the backbone of the pET-Naldt-GG-TALE, excluding the NTR (Supplementary Fig. 7c). The CRD containing 21.5 repeats was assembled into this PCR-amplified product using the fragments with assembled repeats 1–10, 11–20 and 21, and the last half repeat (see ‘Cloning of tSCA21.5 and Naldt-tSCA21.5', above (Supplementary Figure 9c)). All primer and plasmid sequences are available on request.

### Protein purification and labelling

*Protein expression*. BL21 (DE3) electrocompetent *E. coli* cells were co-transformed with plasmids encoding TAL effector constructs and the pBAD-FGE plasmid (generous gift of Dr Taekjip Ha, University of Illinois at Urbana-Champaign). A single colony was grown in 5 ml LB supplemented with 100 μg ml^−1^ ampicillin and 25 μg ml^−1^ kanamycin as a seeding culture until saturation, and subsequently in 500 ml of Terrific Broth at 37 °C and 250 r.p.m. with the corresponding antibiotics until OD_600_ of 0.3–0.4. FGE expression was induced with 0.2% L-arabinose (Sigma) and the culture was grown further at 37 °C and 250 r.p.m. until it reached OD_600_ 0.7–0.8. TAL effector expression was induced with 0.4 mM isopropyl β-D-1-thiogalactopyranoside (Sigma). The proteins were expressed overnight at 16 °C and 250 r.p.m.

*Protein purification*. Cell cultures were centrifuged at 4,000 r.p.m. at 4 °C for 15 min and the pellets were resuspended in 10–20 ml of lysis buffer (25 mM Tris-HCl (Fisher) pH 7.5, 300 mM NaCl (Fisher), 0.5% Triton X-100 (Sigma), 5% glycerol (Sigma), 4 U ml^−1^ DNase I (NEB), 0.3 mM phenylmethanesulfonylfluoride (Sigma), 1 mg ml^−1^ lysozyme (Sigma)). The cells were lysed by sonication for 20 min total with alternating 5 s of pulse and 5 s of rest, and cell debris were centrifuged at 13,000 r.p.m. for 20 min at 4 °C. The His-tagged TAL effectors were purified using AKTA pure chromatography system (GE Healthcare) with a 1 ml HisTrap column (GE Healthcare). The cleared lysate was loaded on the column, washed with 20 mM Tris-HCl pH 7.5, 500 mM NaCl and 20 mM imidazole (Sigma), and eluted with 20 mM Tris-HCl pH 7.5, 500 mM NaCl and 250 mM imidazole. The purified protein was dialysed in 2 l 50 mM phosphate buffer pH 7–8.4, (depending on the predicted protein isoelectric point), 500 mM NaCl at 4 °C overnight. When necessary, the protein was further purified using 16/600 200 pg gel filtration column (GE Healthcare) using 50 mM phosphate buffer pH 7–8.4 (depending on the predicted protein isoelectric point), 500 mM NaCl.

*Protein labelling with Cy3*. The purified (>90% purity by SDS–polyacrylamide gel electrophoresis) TAL effector proteins were buffer exchanged using Amicon Ultra 0.5 ml centrifugal units (EMD Millipore) and concentrated to 100–300 μM in 30 μl of labelling buffer (250 mM potassium phosphate pH 6, 500 mM KCl (Fisher), 5 mM dithiothreitol (Roche)). The concentrated protein solutions were used to resuspend 1 mg Cy3 hydrazide (GE healthcare) and labelled for 24 h at room temperature in the dark. The labelled proteins were diluted with 400 μl fluorescence anisotropy buffer (20 μM Tris-HCl, pH 7.5, 100 mM Nacl, 0.5 mM EDTA (Fisher)) and purified from unreacted Cy3 by two consecutive passages through Micro Bio-spin 6 columns (Bio-Rad) following the manufacturer's instructions.

*Protein labelling with biotin- and streptavidin-coated Qdot705*. The exact protocol followed for Cy3 labelling was utilized, only with the Cy3 hydrazide being replaced with (+)-biotinamidohexanoic acid hydrazide (Sigma). Purified TALE–biotin was conjugated to the streptavidin-coated Qdot705 (Invitrogen) by incubation with 10-fold molar excess Qdots for 15 min.

### Fluorescence anisotropy

A 29-bp oligonucleotide containing the 23 nucleotide TALE-binding site was labelled at the 5′ end with 6-FAM (fluorescein) (IDT) and annealed with its reverse-complementing oligonucleotide in the annealing buffer (10 mM Tris-HCl, 1 mM EDTA, pH 8, 50 mM NaCl) by heating up the mixture at 90 °C for 2 min and gradually cooling to 4 °C (Supplementary Fig. 8). A control double-stranded oligonucleotide was prepared similarly, except the sequence was randomized to contain no binding site (Supplementary Figure 10).

Mixtures of 1 nM double-stranded oligonucleotide and various concentrations of proteins were prepared in the fluorescence anisotropy buffer, and 200 μl samples were assayed in black 96-well plates (Corning), in duplicates. Fluorescence polarization measurements were taken on an Infinite 200 Pro microplate reader (Tecan) using excitation and emission wavelengths of 485 and 535 nm, respectively. The fluorescence polarization values were converted to fluorescence anisotropy values using equation 1, where *A* is anisotropy and *P* is polarization. The *K*_D_ value was calculated by curve fitting on Origin 8.5 using equation 2, where *A* is observed anisotropy value, *A*_f_ is anisotropy of free DNA, *A*_b_ is anisotropy of bound DNA, *L*_T_ is total ligand (DNA) concentration and *R*_T_ is total receptor (protein) concentration.


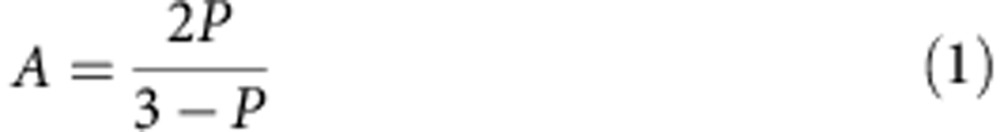



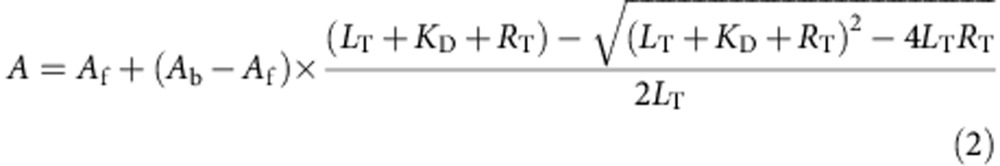


### Flow cell preparation

Mili-fluidic flow cells were constructed by sandwiching two pieces of double-sided tape between a pre-drilled quartz microscope slide and glass coverslip to form a flow channel (∼50 mm long by 4 mm wide by 0.05 mm high). Before assembly of the flow cell, coverslips were functionalized with PEG/PEG–biotin and 0.1 mg ml^−1^ NeutrAvidin (Pierce) for surface attachment of DNA and reduction of non-specific protein adsorption. Polyethylene tubing (PE60, Solomon Scientific) was affixed to the ports (0.048″ OD) drilled in each end of the flow cell to facilitate rapid exchange of buffer solutions and allow for stretching of tethered DNA.

### Surface attachment of double-tethered DNA templates

Assembled flow cells were first incubated with a blocking solution (50 mM MOPS, 10–110 mM KCl, 0.1 mM EDTA, 5% glycerol, 0.3 mg ml^−1^ BSA, pH 8.1) for 10 min and then 5 pM biotin-functionalized DNA for 45 min. Unbound DNA was washed with blocking solution and double-tethered DNA was subsequently formed by flowing 100 nM biotinylated primer, complementary to the single-stranded overhangs previously generated on the long, single-tethered DNA, at a rate of 100 μl min^−1^ in the presence of 100 μM chloroquine (Sigma Aldrich). The presence of chloroquine in the primer solution allows for the DNA to be extended to ∼85% of its contour length immediately before formation of the second surface tether, reducing substrate fluctuations during single-molecule imaging^39^. Chloroquine is subsequently removed by washing the flow cell with blocking solution containing 40 mM MgCl_2_ and 200 mM NaCl for 5 min.

### Single-molecule imaging

Single-molecule imaging experiments were carried out on an inverted microscope (Nikon IX70) equipped for total internal reflection fluorescence coupled with an electron-multiplying charge-coupled device camera (Andor iXon Ultra 897). Cy3-labelled proteins were illuminated using a 532-nm diode-pumped solid-state (DPSS) laser (CrystaLaser). SYTOX green or YOYO-1-labelled DNA was excited using a 488-nm DPSS laser (SpectraPhysics Excelsior). Finally, Qdot705-labelled TALEs were excited using a 637-nm DPSS laser (Coherent). Images were acquired at a rate of 30–50 Hz. Dye-labelled TALE proteins were added at concentrations typically ranging from 25 to 100 pM, and in the case of the NTD, in the presence of 1–2 nM unlabelled constructs. For quantum dot-conjugated TALEs, 100–200 pM labelled TALEs were incubated in a prepared sample chamber, followed by a rinse with 150 mM KCl and introduction of 2 nM YOYO-1 (Invitrogen) in imaging buffer. Five micromolar of free biotin (Sigma) was also added during Qdot experiments to minimize non-specific binding of Qdot–TALE conjugates to the chamber surface. A reducing agent (7 mM β-mercaptoethanol, Sigma Aldrich) and oxygen-scavenging system (glucose oxidase and bovine liver catalse) along with 1% glucose by volume were added to the buffer, along with the protein.

### Data analysis

Data was recorded as multi-image stacks in TIF (Tagged Image File) format using the Andor Solis software. Regions of interest containing single-diffusing TALE proteins are isolated using ImageJ, and the centroid locations of single proteins are determined using RapidStorm fitting software^63^. Single-molecule trajectories were then further analysed using custom MATLAB scripts, available from the authors on request.

### Localizing single-TALE proteins via fluorescence microscopy

Error in localizing single-TALE proteins arises from two main sources as follows: (1) uncertainty due to the finite number of photons collected within each frame during image acquisition, which amounts to localization precision and (2) thermal fluctuations in the DNA template parallel to the direction of TALE diffusion, which amounts to localization accuracy. For these experiments, we use an acquisition time of 33 ms per frame, during which we collect ∼70–100 photons. We note that the imaging assay also shows an extremely low fluorescence background. On the basis of these experimental conditions, we estimate the localization precision of single-TALE proteins to be ∼25 nm (ref. 44). Regarding the second source of error, we estimate the magnitude of DNA fluctuations parallel to the direction of TALE diffusion to be ∼40 nm, which is based on the equipartition theorem and a Taylor series expansion of the DNA stretching force under experimental conditions. This calculation relies on the average extension of DNA templates in our assay (∼90% of full contour length) and the Marko–Siggia force-extension relation^64^. The summation of both sources of error is ∼65 nm.

### CVE for 1D diffusion coefficients

To minimize compounding localization errors of each frame in a single-molecule trajectory, which is inherently present when using MSD determination of diffusion coefficients, we utilized a CVE to determine 1D diffusion coefficients^45^. Using an average displacement *Δx*_*n*_ of a TALE protein during a single acquisition period (33 ms per frame) and the localization error *<δz*^*2*^*>* as described above, 1D diffusion coefficients were determined according to:


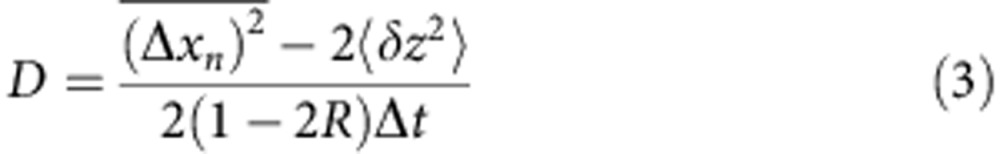


Where *R=1/6* is a motion blur coefficient accounting for the finite exposure time of the camera, which corresponds to the camera shutter being open for the entire time *Δt* an image is recorded.

### Hydrodynamic models for TALE diffusion

In order to estimate the effects of hydrodynamic friction on TALEs with increasing numbers of central repeats, we utilized a model developed for a rigid helix diffusing along its long axis^56^. The MSD of such a helix is given as:


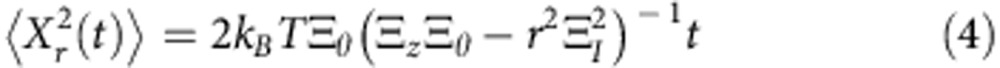


With coefficients of friction given as:


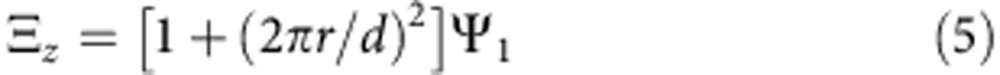



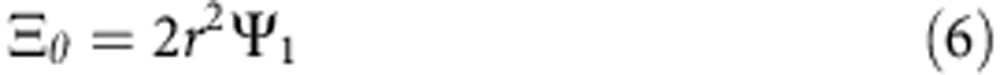



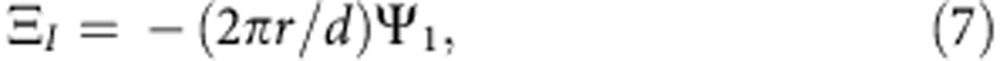


where *d* is the pitch of the helix and *r* is the radius of the helix, and


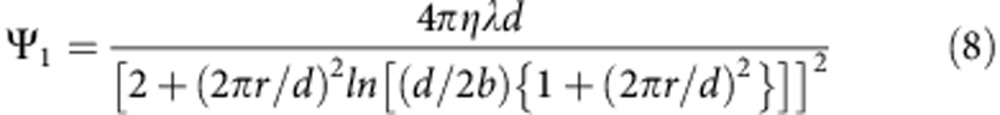


where *b* is the radius of the helical element and *λ* is the number of pitches in the TALE protein. This model predicts a *λ*^−1^ scaling for the diffusion coefficient of TALEs as a function of the number of repeats in the entire helical structure.

We further considered a model for rotationally coupled 1D diffusion of DNA-binding proteins proposed by Blainey and co-workers^48^ to examine the effects of TALE repeat length on 1D diffusion. In this model, a protein is modelled as a sphere that tracks the helical structure of DNA, wherein one portion of the protein (the ‘reading' domain) maintains constant contact with the DNA such that this domain is able to continually interact with the nucleobases for sequence checking. Here we considered the TALE NTR as the ‘reading' domain that maintains contact with the DNA, while the CRD+CTR is considered as a globular protein that is pulled along by the NTR during the ‘search' mode. In essence, this model differs from the helical pitch model (above) in that during the non-specific search mode, the CRD+CTR domain is assumed to adopt a globular structure instead of a highly ordered helical structure. On transitioning to the ‘check' mode, the protein would undergo a major conformation change for local sequence checking.

We estimated the hydrodynamic radius of CRD+CTR domains using a common scaling formula for the size of a globular protein^65^:


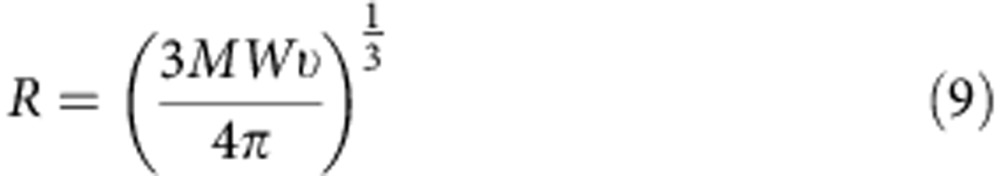


where *MW* is the molecular weight of the domain and *ν* is the specific gravity.

Next, we obtained the scaling of 1D diffusion with protein size as:


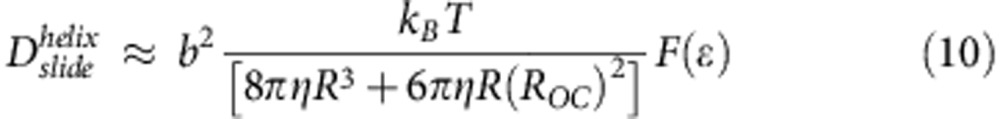


where *b* is the helical pitch of DNA and *R* and *R*_OC_ are the protein radius and minimum distance from the protein center to DNA center, respectively. Here *F(ɛ)* describes the energy landscape experienced along the DNA helix by the diffusing protein, which is assumed to be small (∼1 *k*_B_*T*), as discussed in the main text. Using estimates from the available crystal structures, we approximate *R*_*OC*_≈*R*+1 nm. We obtain a scaling for 1D diffusion based on variations in protein size given by:


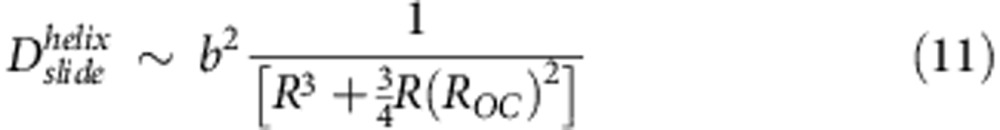


## Additional information

**How to cite this article:** Cuculis, L. *et al.* Direct observation of TALE protein dynamics reveals a two-state search mechanism. *Nat. Commun.* 6:7277 doi: 10.1038/ncomms8277 (2015).

## Supplementary Material

Supplementary Information Supplementary Figures 1-10

## Figures and Tables

**Figure 1:**
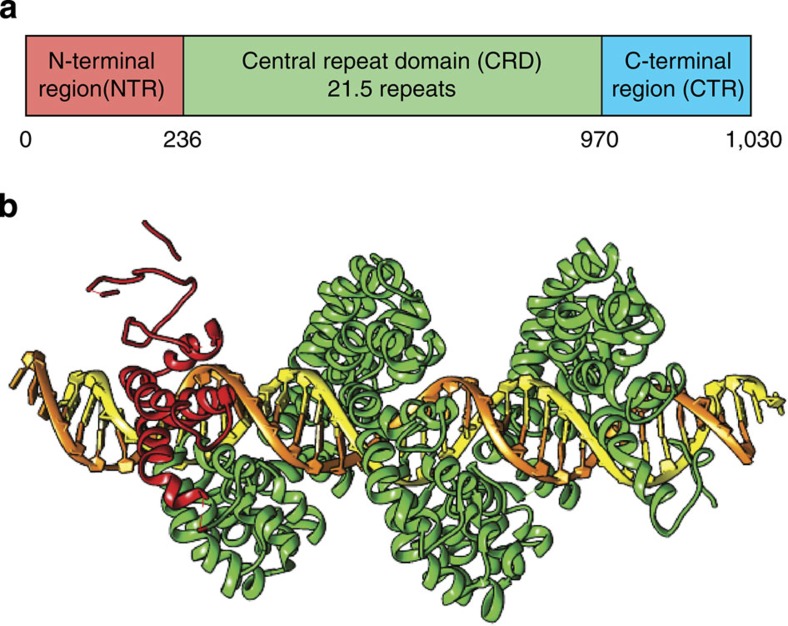
TALE protein structure and subdomain schematic. (**a**) General schematic of the TALE polypeptide chain. The N-terminal region (NTR) contains the type III translocation system necessary for secretion, the central repeat domain (CRD) contains the conserved 34 amino-acid repeats for sequence-specific DNA-binding and the C-terminal region contains nuclear localization signals and an acidic activation domain. (**b**) Co-crystal structure of the PthXo1 TALE bound to its specific DNA target^22^, with only the CRD region and a portion of the NTR shown for display^66^.
