# Supplementary material for: Direct observation of TALE protein dynamics reveals a two-state search mechanism
Source: Nat Commun. 2015 Jun 1;6:7277. doi: 10.1038/ncomms8277 (PMC4458887; doi:10.1038/ncomms8277)
Supplement: Supplementary Information — Supplementary Figures 1-10 [file ncomms8277-s1.pdf]

Supplementary Information

Direct Observation of TALE Protein Dynamics Reveals a Two-state Search Mechanism

Luke Cuculis[1*], Zhanar Abil[2*], Huimin Zhao[1,2,3,4,5],
and Charles M. Schroeder[1,3,4,5]

[1] Department of Chemistry, University of Illinois at Urbana-Champaign
[2] Department of Biochemistry, University of Illinois at Urbana-Champaign
[3] Department of Chemical & Biomolecular Engineering, University of Illinois at Urbana-Champaign
[4] Center for Biophysics and Quantitative Biology, University of Illinois at Urbana-Champaign
[5] Institute for Genomic Biology, University of Illinois at Urbana-Champaign
[*] These authors contributed equally to this work.
Correspondence should be addressed to H.Z. (zhao5@illinois.edu) and C.M.S. (cms@illinois.edu)

Supplementary Text and Figures

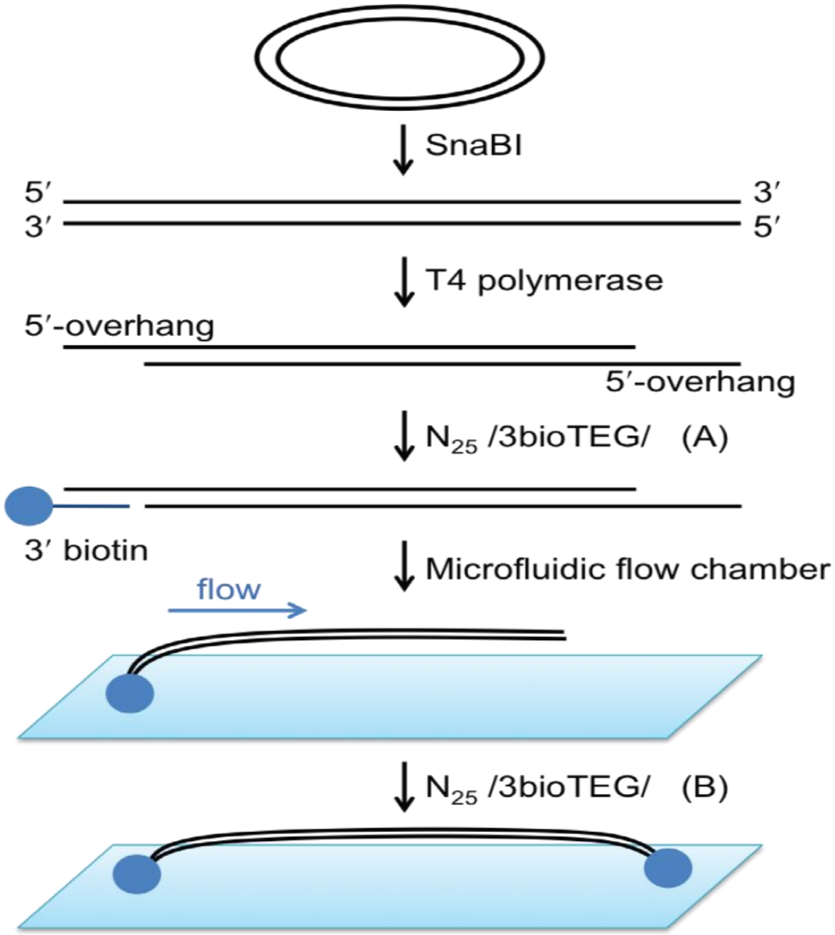

Supplementary Figure 1: Dual-tethered single molecule assay for TALE protein diffusion. First, the large DNA plasmid is linearized by cleavage at a single site with the SnaBI restriction endonuclease. Next, the 3’-5’ exonuclease activity of T4 DNA polymerase is used to create the 5’ overhangs at both ends of the linearized DNA. The exposed 5’-overhangs are used to sequence specifically anneal 3’-biotinylated oligonucleotides. In this process, oligonucleotide A is first annealed to the substrate DNA, followed by attaching the DNA to the surface of the microscope coverslip of the flow chamber through a biotin-NeutrAvidin linkage. Oligonucleotide B is then introduced to the chamber and annealed to the second 5’-overhang, which results in the attachment of the second end of the substrate DNA.

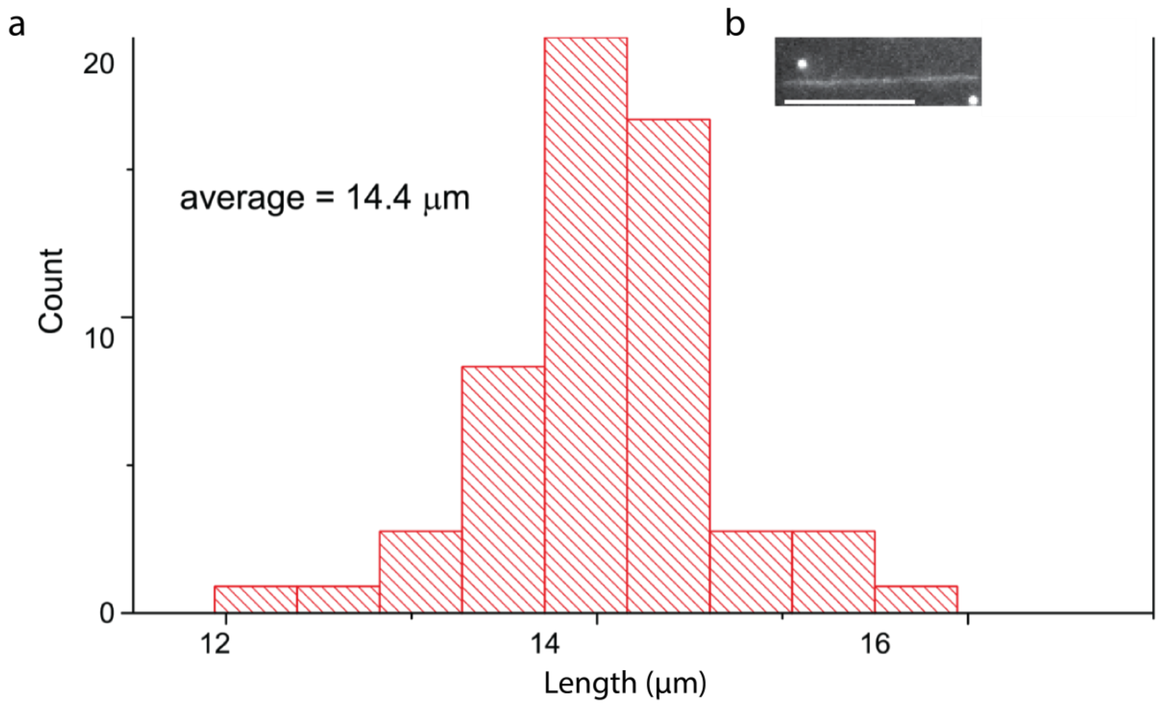

Supplementary Figure 2: Distribution of end-to-end stretched length of dual-tethered DNA templates. (a) Distribution of sizes of stretched, dual-tethered DNA templates. The 44.5 kbp substrates are stretched to an average of 14.4 μm. (b) Representative image of SYTOX Green labeled, dual-tethered DNA molecules. The number of molecules measured, *n,* is equal to 83. Scale bar = 10 μm.

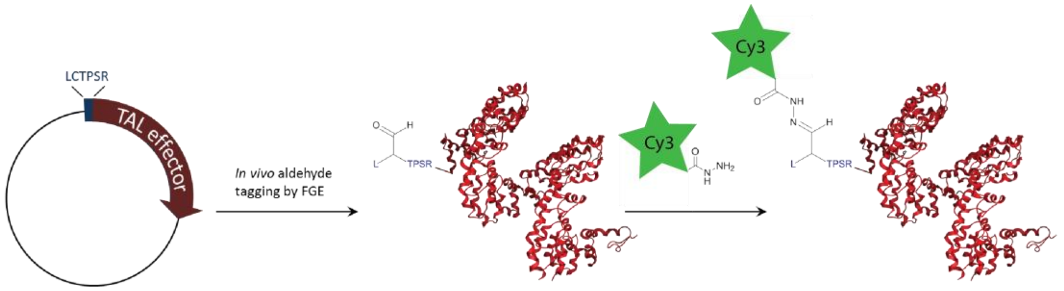

Supplementary Figure 3: Aldehyde labeling scheme used to tag TALE proteins with Cy3 dye at the N-terminus in a 1:1 dye:protein stoichiometry. A six amino acid motif (LCTPSR) is cloned into the N-terminus of the TALE protein, and the construct is co-expressed with formylglycine generating enzyme (FGE) that then converts the cysteine to a formylglycine bearing an unnatural aldehyde. Aldehyde-specific conjugation via hydrazine-functionalized Cy3 organic dyes then allows for non-perturbative, site-specific labeling.

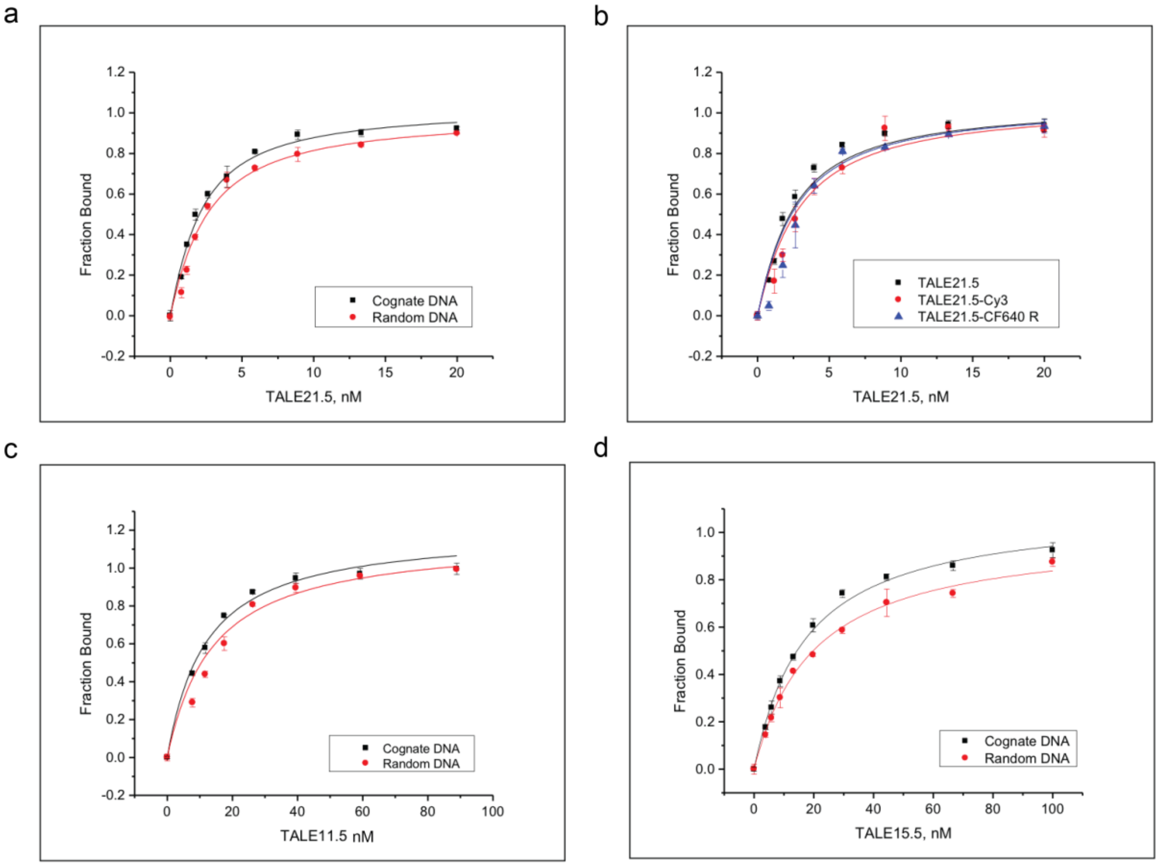

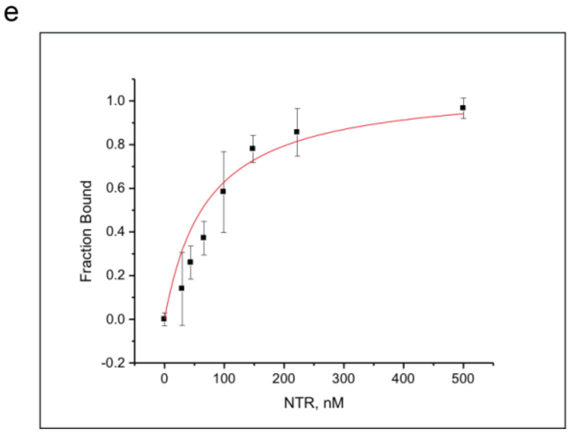

f

Construct	K_d with cognate target, nM	K_d with random target, nM
TALE21.5	1.5 ± 0.3	2.3 ± 0.3
TALE15.5	16.7 ± 0.2	19.3 ± 0.3
TALE11.5	11.1 ± 2.0	13. ± 3.4
NTR	ND	70 ± 27

Supplementary Figure 4: Fluorescence polarization data for TALE constructs used in single molecule experiments. (a) Data for unlabeled TALE with a 21.5 repeat CRD binding to target and random DNA. (b) Data for the 21.5 repeat unlabeled TALE, the TALE with aldehyde and Cy3 labels, and with the aldehyde tag and CF643R. (c) Data for the aldehyde tagged 11.5 repeat TALE binding to target and random DNA. (d) Data for the aldehyde tagged 15.5 repeat TALE binding to target and random DNA. (e) Data for the TALE NTR truncation with the aldehyde tag binding to random DNA. (f) Summary of dissociation constants (K_d) for the four TALE constructs.

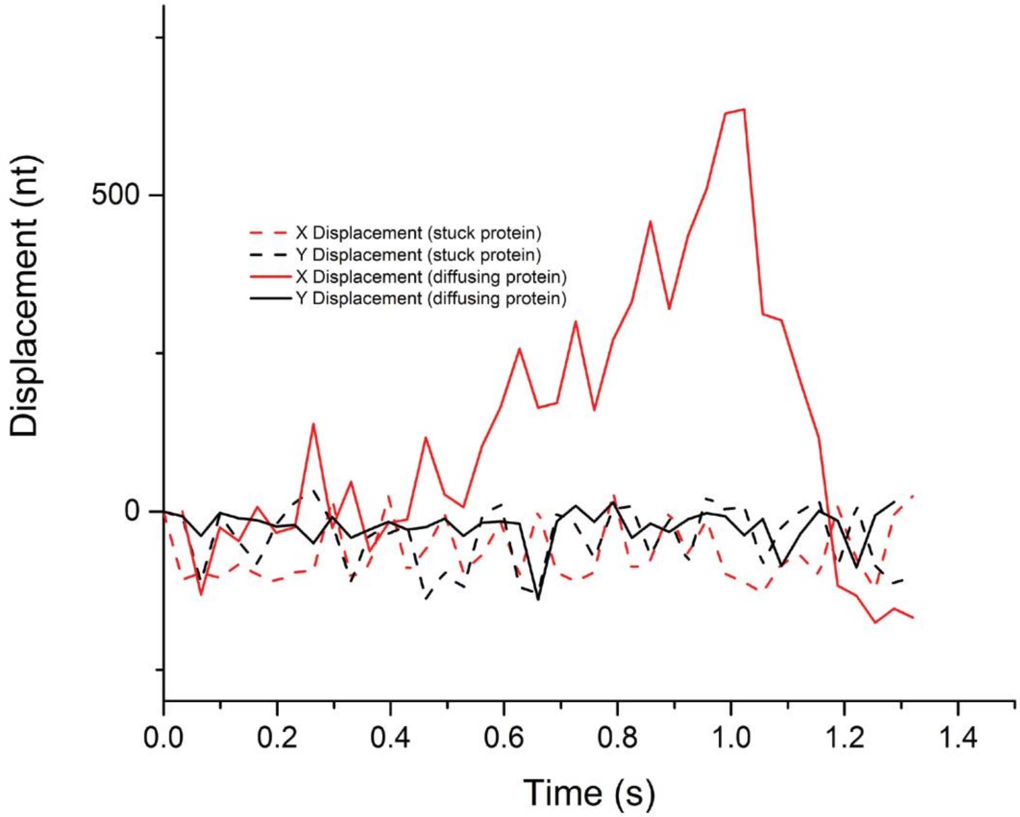

Supplementary Figure 5: Representative displacements for a single TALE diffusing along a DNA template (solid lines) in the transverse (x-direction, along DNA) and perpendicular (y-direction, orthogonal to DNA) directions. The displacement of the TALE perpendicular to the DNA backbone is on the order of the apparent fluctuations of a stationary TALE, in contrast to the displacement of the TALE along the DNA backbone that is governed by 1-D diffusion. For reference, the displacement of a TALE protein immobilized on the coverslip surface (dashed lines) is shown.

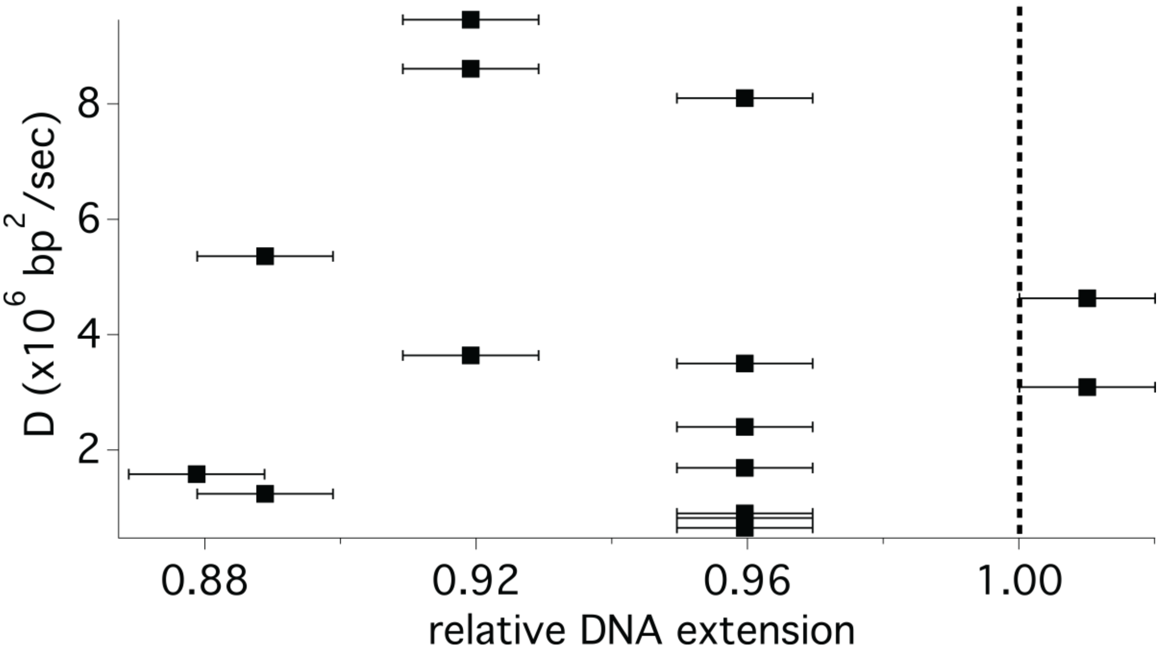

Supplementary Figure 6. Apparent 1D diffusion coefficients for single TALE binding events plotted against the relative extension (L/L_0) of the DNA substrates on which the binding events were observed. No correlation between relative extension and diffusion coefficient is observed and error bars are the result of the +/- 1 pixel accuracy with which DNA extension can be measured. The dashed line indicates the predicted crystallographic length of the substrate as B-form DNA. Due to the use of chloroquine, an intercalating agent, during DNA extension and tethering, this length is occasionally surpassed for dual-tethered templates.

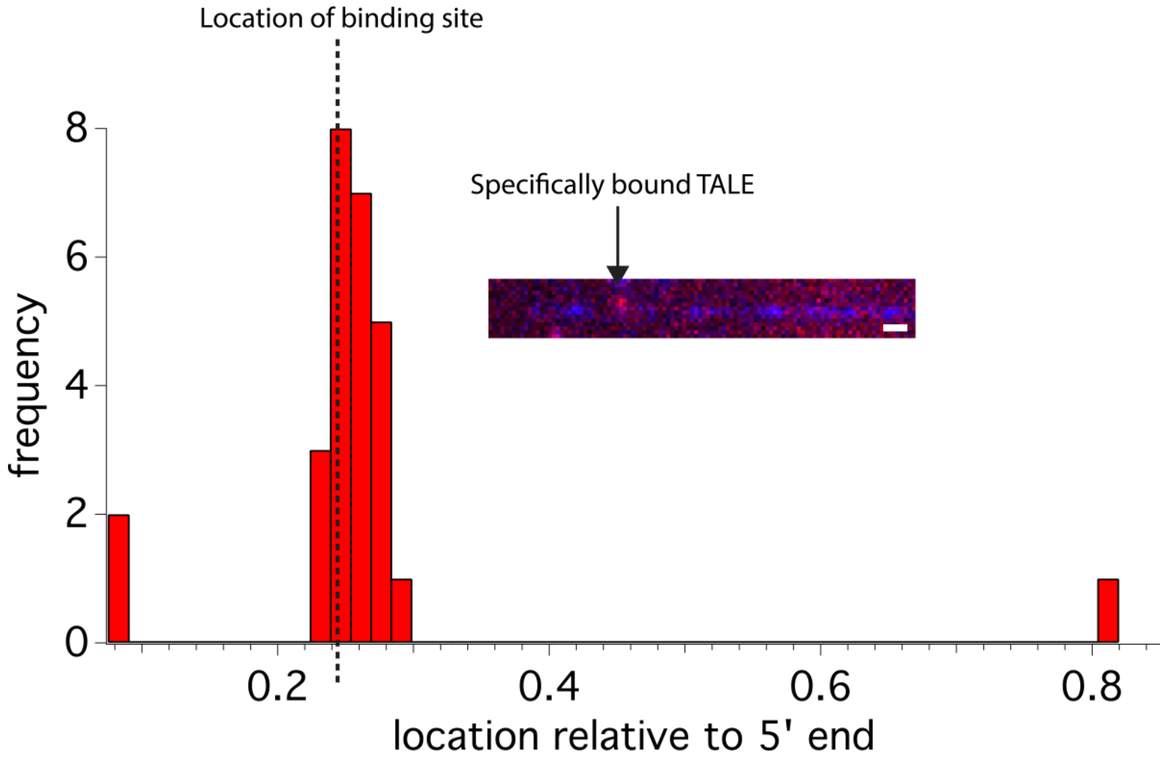

Supplementary Figure 7. Observed site-specific, stable binding of Qdot705 labeled TALEs. The frequency of observed stable binding events is plotted against their location relative to the 5' end of the DNA substrate. The location of the binding site (~11kb from the 5' end) is indicated by the dashed line. A representative co-localized image of DNA (blue, YOYO1 stained DNA) and specifically-bound TALE (red, Qdot705 TALE) is shown (inset). Scale bar = 1 μm.

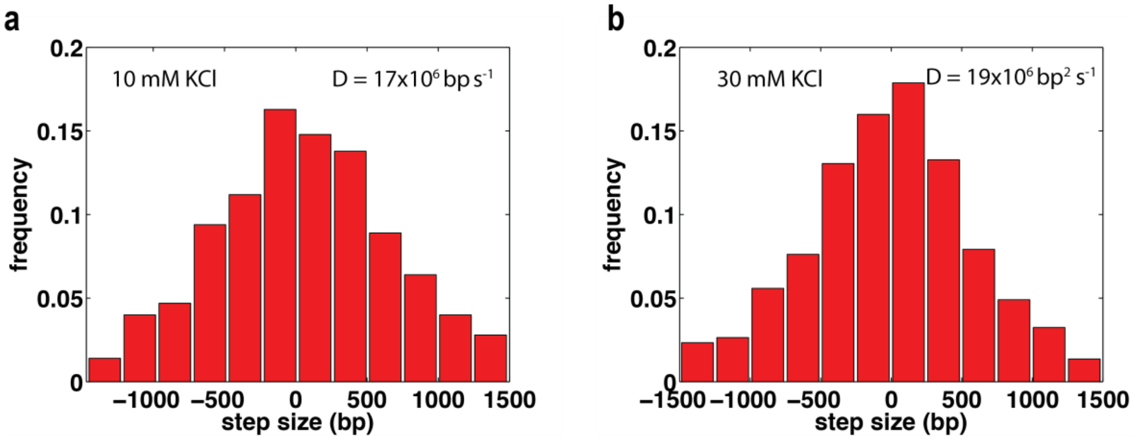

Supplementary Figure 8: Distributions of TALE NTR step size (33 ms frame rate) at (a) 10 mM KCl and (b) 30 mM KCl. For each data set, the number of total trajectories analyzed is n = 50.

a

MGPLCTPSRSSHHHHHHSSGLVPRGSHMLDTSLLDSMPAVGTPHTAAAPAECDEVQSGLRAADDPPPTVRVAVTA
ARPPRAKPAPRRRAAQPSDASPAAQVDLRTLGYSQQQQEKIKPKVRSTVAQHHEALVGHGFTHAHIVALSQHPAA
LGTVAVTYQDIIRALPEATHEDIVGVGKQWSGARALEALLTEAGELRGPPLQLDTGQLLKIAKRGGVTAVEAVHA
WRNALTGAPLNLTPDQVVAIASNIGGKQALETVQRLLPVLCQDHGLTPDQVVAIASNNGGKQALETVQRLLPVLC
QDHGLTPDQVVAIASHDGGKQALETVQRLLPVLCQDHGLTPDQVVAIASNIGGKQALETVQRLLPVLCQDHGLTP
DQVVAIASNIGGKQALETVQRLLPVLCQDHGLTPDQVVAIASHDGGKQALETVQRLLPVLCQDHGLTPDQVVAIA
SHDGGKQALETVQRLLPVLCQDHGLTPDQVVAIASNGGGKQALETVQRLLPVLCQDHGLTPDQVVAIASHDGGKQ
ALETVQRLLPVLCQDHGLTPDQVVAIASNIGGKQALETVQRLLPVLCQDHGLTPDQVVAIASNIGGKQALETVQR
LLPVLCQDHGLTPDQVVAIASNIGGKQALETVQRLLPVLCQDHGLTPDQVVAIASHDGGKQALETVQRLLPVLCQ
DHGLTPDQVVAIASNIGGKQALETVQRLLPVLCQDHGLTPDQVVAIASNNGGKQALETVQRLLPVLCQDHGLTPD
QVVAIASNIGGKQALETVQRLLPVLCQDHGLTPDQVVAIASHDGGKQALETVQRLLPVLCQDHGLTPDQVVAIAS
NIGGKQALETVQRLLPVLCQDHGLTPDQVVAIASHDGGKQALETVQRLLPVLCQDHGLTPDQVVAIASHDGGKQA
LETVQRLLPVLCQDHGLTPDQVVAIASNIGGKQALETVQRLLPVLCQDHGLTPDQVVAIASNGGGKQALESIVAQ
LSRPDPALAALTNDHLVALACLGGRPALDAVKKGLPHAPELIRRINRRIPERTSHRVA

b

MGPLCTPSRSSHHHHHHSSGLVPRGSHMLDTSLLDSMPAVGTPHTAAAPAECDEVQSGLRAADDPPPTVRVAVTA
ARPPRAKPAPRRRAAQPSDASPAAQVDLRTLGYSQQQQEKIKPKVRSTVAQHHEALVGHGFTHAHIVALSQHPAA
LGTVAVTYQDIIRALPEATHEDIVGVGKQWSGARALEALLTEAGELRGPPLQLDTGQLLKIAKRGGVTAVEAVHA
WRNALTGAPLN

c

MGLCTPSRSSHHHHHHSSGLVPRGSHMLDTSLNLTPDQVVAIASNIGGKQALETVQRLLPVLCQDHGLTPDQVVA
IASNNGGKQALETVQRLLPVLCQDHGLTPDQVVAIASHDGGKQALETVQRLLPVLCQDHGLTPDQVVAIASNIGG
KQALETVQRLLPVLCQDHGLTPDQVVAIASNIGGKQALETVQRLLPVLCQDHGLTPDQVVAIASHDGGKQALETV
QRLLPVLCQDHGLTPDQVVAIASHDGGKQALETVQRLLPVLCQDHGLTPDQVVAIASNGGGKQALETVQRLLPVL
CQDHGLTPDQVVAIASHDGGKQALETVQRLLPVLCQDHGLTPDQVVAIASNIGGKQALETVQRLLPVLCQDHGLT
PDQVVAIASNIGGKQALETVQRLLPVLCQDHGLTPDQVVAIASNIGGKQALETVQRLLPVLCQDHGLTPDQVVAI
ASHDGGKQALETVQRLLPVLCQDHGLTPDQVVAIASNIGGKQALETVQRLLPVLCQDHGLTPDQVVAIASNNGGK
QALETVQRLLPVLCQDHGLTPDQVVAIASNIGGKQALETVQRLLPVLCQDHGLTPDQVVAIASHDGGKQALETVQ
RLLPVLCQDHGLTPDQVVAIASNIGGKQALETVQRLLPVLCQDHGLTPDQVVAIASHDGGKQALETVQRLLPVLC
QDHGLTPDQVVAIASHDGGKQALETVQRLLPVLCQDHGLTPDQVVAIASNIGGKQALETVQRLLPVLCQDHGLTP
DQVVAIASNGGGKQALESIVAQLSRPDPALAALTNDHLVALACLGGRPALDAVKKGLPHAPELIRRINRRIPERT
SHRVA

Supplementary Figure 9. Amino acid sequences of TALE constructs used in this study. (a) 21.5 repeat TALE with NTR and CTR regions. (b) TALE NTR only. (c) TALE CRD+CTR only.

Cognate DNA sequence

/56-FAM/5′-ATCTAGCAACCTCAAACAGACACCATACG-3′

3′-TAGATCGTTGGAGTTTGTCTGTGGTATGC-5′

Random DNA sequence

/56-FAM/5′-ATCAGACCGACATCTAATCCGCAACACAA-3′

3′-TAGTCTGGCTGTAGATTAGGCGTTGTGTT-5′

Supplementary Figure 10. DNA sequences of target and random double stranded oligonucleotides used in fluorescence polarization experiments. Note that the abbreviation /56-FAM/ corresponds to 5’ 6-FAM, which is a single isomer derivative of fluorescein.
